# CXCL1 Contributes to β-Amyloid-Induced Transendothelial Migration of Monocytes in Alzheimer’s Disease

**DOI:** 10.1371/journal.pone.0072744

**Published:** 2013-08-14

**Authors:** Ke Zhang, Li Tian, Li Liu, Yu Feng, Yan-Bin Dong, Bo Li, De-Shu Shang, Wen-Gang Fang, Yun-Peng Cao, Yu-Hua Chen

**Affiliations:** 1 Department of Developmental Cell Biology, Key Laboratory of Cell Biology, Ministry of Public Health, Key Laboratory of Medical Cell Biology, Ministry of Education, China Medical University, Shenyang, China; 2 Department of Neurology, the First Affiliated Hospital, China Medical University, Shenyang, China; Massachusetts General Hospital and Harvard Medical School, United States of America

## Abstract

**Background:**

Bone marrow-derived microglia that originates in part from hematopoietic cells, and more particularly from monocytes preferentially attach to amyloid deposition in brains of Alzheimer’s disease (AD). However, the mechanism of monocytes recruited into the amyloid plaques with an accelerated process in AD is unclear.

**Methodology/Principal Findings:**

Here we reported that monocytes from AD patients express significantly higher chemokine (C-X-C motif) ligand 1 (CXCL1) compared to age-matched controls. AD patient’s monocytes or CXCL1-overexpressing THP-1 cells had enhanced ability of β-amyloid (Aβ)-induced transendothelial migration and Aβ-induced transendothelial migration for AD patient’s monocytes or CXCL1-overexpressing THP-1 cells was almost abrogated by anti-CXCL1 antibody. Furthermore, monocytes derived from a transgenic mouse model of AD also expressed significantly higher CXCL1. CD11b^+^CD45^hi^ population of cells that were recruited from the peripheral blood were markedly bolcked in APP mouse brain by anti-CXCL1 antibody. Accordingly, in response to Aβ, human brain microvascular endothelial cells (HBMEC) significantly up-regulated CXC chemokine receptor 2 (CXCR2) expression, which was the only identified receptor for CXCL1. In addition, a high level expression of CXCR2 in HBMEC significantly promoted the CXCL1-overexpressing THP-1 cells transendothelial migration, which could be was abrogated by anti-CXCR2 antibody. Further examination of possible mechanisms found that CXCL1-overexpressing THP-1 cells induced transendothelial electrical resistance decrease, horseradish peroxidase flux increase, ZO-1 discontinuous and occludin re-distribution from insoluble to soluble fraction through interacting with CXCR2. ROCK inhibitor, Y27632, could block CXCL1-overexpressing THP-1 cells transendothelial migration, whereas other inhibitors had no effects.

**Conclusions/Significance:**

The present data indicate that monocytes derived from AD patients overexpressing CXCL1, which is a determinant for Aβ-induced transendothelial migration. CXCL1 expressed by monocytes and CXCR2 on HBMEC is involved in monocytes migrating from blood to brain in AD patients.

## Introduction

Alzheimer’s disease (AD) is the most common form of age-related dementia [1]. The pathological hallmarks of AD are senile plaques composed of beta-amyloid (Aβ), intracellular neurofibrillary tangles, accumulation of activated microglia and astrocytes around Aβ plaques, and degenerative neurons [2,3].

Mounting evidence has demonstrated that microglia as the immune cells of the central nervous system (CNS) [4,5], which are responsible for patrolling the brain micro-environment and responding quickly in the presence of pathogens and brain damages, accumulate and surround the senile plaques in brains of post-morten AD patients and rodent transgenic models of AD [4–9]. The exact role for microglia in the pathogenesis of AD remains to be elucidated. One first proposal is that microglia can be activated by Aβ and express receptors such as class A scavenger receptor, CD36 and RAGE to promote phagocytosis of Aβ [10,11]. Microglia also secrete proteolytic enzymes (insulin-degrading enzyme, neprilysin and matrix metalloproteinase 9) that may eliminate Aβ deposition and limit AD process [12,13], thus performing a neuroprotective role in AD. On the other hand, activated microglia by Aβ can also produce cytokines, chemokines and neurotoxins that may promote neuronal degeneration [14–17].

Two different types microglia exist in the brain, the resident and newly differentiated microglia that are derived from bone marrow [4,18–21]. Bone marrow-derived microglia originates in part from hematopoietic cells, and more particularly from monocytes [4,22,23]. In normal adult brains, a limited number of mononuclear phagocytes are continuously recruited into the brain, whereas recent data from animal models of AD suggest that bone marrow-derived microglia are recruited into the amyloid plaques with an accelerated process [24–27]. Of our interest, bone marrow-derived microglia express higher levels of proteins that are required for antigen presentation and thus are more efficient in restricting amyloid burden and preventing the formation or eliminating Aβ plaques deposition than resident counterparts [28–30]. However, the mechanism of bone marrow-derived microglia crossing blood-brain barrier (BBB) in AD is not well-understood. Recently we investigated the transcriptional differences between peripheral blood monocytes of AD patients and aged matched controls to identify the determinants contributing to monocytes migrating from blood into brain in AD. Our results show that monocytes derived from AD patients over-express Chemokine (C-X-C motif) ligand 1 (CXCL1) that interact with CXC chemokine receptor 2 (CXCR2) in HBMEC to facilitate Aβ-induced transendothelial migration through the endothelial tight junction.

## Material and Methods

### Cell culture

Human brain microvascular endothelial cells (HBMEC) were a generous gift from Dr. K.S. Kim (Johns Hopkins University, Baltimore, MD) [31,32]. They were cultured in RPMI 1640 medium, supplemented with 10% fetal bovine serum (FBS; Hyclone, Logan，UT), 10% Nu-serum (BD Biosciences, Bedford，MA), 2 mM glutamine, 1 mM sodium pyruvate, 1 × non-essential amino acid and 1 × minimal essential medium (MEM) vitamin. Human acute monocytic leukemia cell line THP-1 (ATCC, Rockville, MD) was maintained in RPMI 1640 medium, supplemented with 10% FBS. 293T (ATCC, Rockville, MD) were cultured in Dulbecco’s modiﬁed Eagle’s medium (DMEM; Mediatech, Herndon, VA) with 10% FBS. All cells were incubated at 37° C in a 5% CO_2_, 95% air humidified atmosphere.

### Human subjects

Subjects with AD were collected from the First and Second Afﬁliated Hospital, China Medical University, under an International Review Board-approved human studies protocol. The patients with AD have no immunological diseases and vascular risk factors such as hypertension, cardiac disease, and diabetes. The diagnosis of AD was based on National Institute of Neurological Disorders, Stroke-Alzheimer Disease and Related Disorders Association criteria [33] and included use of the Mini Mental State Exam [34]. None of the subjects had experienced infection or taken immuno-regulation drugs during the period of 6 month before sample collection. Overall, 23 patients with AD (13 males and 10 females, aged 60–88 years, mean age 75.3±3.5 years) and 21 age-matched control individuals (10 males and 11 females, mean age 70.6±8.6 years) were evaluated in this study. The next of kin or guardians for all the participants provided their written informed consent to participate in this study. Above studies and consent procedure have been reviewed and approved by China Medical University Review Committee.

### Transendothelial migration assay of monocytes

Human peripheral monocytes were separated with Dynabeads Isolation Reagent (Invitrogen, Carlsbad, CA) according to the manufacturer’s protocols. Monocytes transendothelial migration was performed as described previously [35]. Briefly, 2 × 10^5^ HBMEC were seeded on the upper chamber of Transwell insert with 5 μm pore size (Corning Costar Corp., Cambridge, MA) in 24-well plates. The integrity of the HBMEC monolayer was monitored by daily transendothelial electrical resistance (TEER) measurements, using a Millicell-ERS endothelia volt-ohmmeter (World Precision Instruments Inc.). Experiments were conducted 4d after plating when TEER was > 200 Ω × cm^2^. Monocytes (1 × 10^6^) from AD patient or elderly control or THP-1 cells were loaded into the upper chamber of the Transwell insert and Aβ (125nM) was added to the lower chamber. After incubation for 8 or 24h, migrated monocytes from the lower chamber were collected and counted in a hemacytometer in triplicate. In some experiments, anti-CXCL1 or anti-CXCR2 neutralization antibody (R&D Systems) was added to the upper chamber. After incubation for 24h, the cells that had transmigrated into the lower chamber were harvested and counted in a hemocytometer.

### Real-time reverse transcription (RT)-PCR

The total RNA isolated with TRIzol reagent (Invitrogen, Carlsbad, CA) was reverse transcribed using Moloney murine leukemia virus (M-MLV) reverse transcriptase (Promega, Madison, WI). Real-time PCR was performed on an ABI 7500 Real-time PCR system (Applied BioSystems) with a SYBR premix Ex *Taq* Kit (Takara Biotechnology, Dalina, China), according to the manufacturer’s instructions. The primer sequences for human CXCL1 were ACTGCTGCTCCTGCTCCT (forward) and CGATGATTTTCTTAACTATGGG (reverse); primer sequences for mouse CXCL1 were AGACCATGGCTGGGATTCAC (forward) and CTCGCGACCATTCTTGAGTGT (reverse); primer sequence for human CXCR2 was TGCGGGTCATCTTTGCTGTC (forward) and GAGCCCGGTCGATGTGATTG (reverse). GAPDH (glyceraldehyde-3-phosphate dehydrogenase) was chosen as internal controls and the primers for GAPDH were described previously [31]. The ampliﬁcation conditions were as follows: 95° C for 10 s, and 40 cycles of 95° C for 5 s and 60° C for 34 s. The comparative cycle threshold (*C*_*T*_) method was used to calculate the relative gene expression level, with GAPDH as the internal control. Real-time PCR products were analyzed on agarose gel electrophoresis and were veriﬁed by DNA sequencing.

### Animals and experimental groups

APP/PS1 double transgenic mice (B6C3-Tg (APPswe, PSEN1dE9) 85Dbo/J mice) were used in the present study (breeding pairs were obtained from the Jackson Laboratory, West Grove, PA). They were kept in cages in a controlled environment (22–25°C, 50% humidity). Mice at the age of 9 months were randomly assigned to one of two groups: anti-CXCL1 group, 200 μl anti-CXCL1 polyclonal antibody (150 μg/ml) were injected i.p. at the 1st and the 4th day; isotypic IgG group, the same concentration of isotypic IgG were injected i.p. at the 1st and the 4th day. Mice were sacrificed at the 7th day. Blood samples were collected from heart and monocytes were isolated by the Ficoll-Hypaque gradient technique. The experimental procedures were carried out in accordance with the regulations of the animal protection laws of China and approved by the animal ethics committee of China Medical University (JYT-20060948). All efforts were made to minimize animal suffering and the number of animals used.

### Staining brain cells for CD11b and CD45 for ﬂow cytometry

Staining brain cells were performed as described previously [26,36]. Briefly, fresh brain cells were prepared followed by percoll gradient centrifugation and were incubated with allophycocyanin (APC)-labeled antibodies to CD45 (1.25 μg/ml) and/or phycoerythrin (PE)-labeled antibodies to CD11b (1.25 μg/ml) (both from eBioscience, USA), or with isotype-matched control antibodies, for 30 min on ice. Fluorescence intensity was measured using a FACScalibur (BD) ﬂow cytometer.

### Cell fractionation and Western Blotting

Conﬂuent HBMEC were washed with Dulbecco’s phosphate-buffered saline containing 0.1 mM EDTA without calcium and magnesium for three times, extracted in TritonX-100 lysis buffer (25 mM HEPES, 150 mM NaCl, 4 mM EDTA, 1% TritonX-100, protease inhibitors), centrifuged at 14,000×g for 10 min. The supernatant was collected as the soluble fraction. The pellets were dissolved in SDS lysis buffer (1% SDS, 25 mM HEPES, 4 mM EDTA, protease inhibitors) to eliminate insoluble material. The total cell lysates were prepared with radioimmunoprecipitation assay (RIPA) buﬀer (50 mM Tris-HCl, 150 mM NaCl, 1% NP-40, 0.5% deoxycholate, 0.1% sodium dodecylsulfate, protease inhibitors). Equal amounts of proteins were separated by SDS-PAGE and processed for immunoblotting with antibodies for occludin (Zymed Laboratories, Inc, South San Francisco, CA) or CXCR2 (Abcam, HKSTP, Hong Kong). Immune complexes were visualized using SuperSignal Chemiluminescence substrates (Pierce, Indianapolis, IN). Immunoreactive bands were visualized by Super Signal West Pico chemiluminescent substrate (Pierce, Rockford, IL) by using LAS3000mini (Fuji Film, Tokyo, Japan). For quantitative analysis, the mean density of each band was measured by Multi Gauge V 3.1 software, and the band density of the activated form of the protein was divided by the density of the corresponding total protein band to obtain the normalized band density. Data were plotted as percentages of the control.

### Lentiviral vector-mediated over-expression of CXCL1 in THP-1 cells

#### (i) Lentiviral vector construction and lentivirus production

The full-length open reading frame (ORF) of CXCL1 was subcloned into lentivirus expression vector pCDH cDNA Cloning and Expression Lentivectors (SBI, Mountain View, CA, CD513B-1). The primer sequences for CXCL1 were TGC*TCTAGA*CGCCACCATGGCCCGCGCTGCTCTC (forward) and CGC*GGATCCT*CAGTTGGATTTGTCACTGTTC (forward). The lentiviral vector particles were produced by Lipofectamine 2000 (Invitrogen, Carlsbad, CA) transfection into 293T cells as previously described [37,38]. Briefly, 1 × 10^6^ 293T cells were seeded in a 10 cm tissue dishes 24 hours prior to transfection. A total of 16μg plasmids DNA were used for the transfection as 2μg pMD2.G, 6μg pspax2 packaging plasmids and 8μg constructed lentiviral vectors containing full-length ORF of CXCL1. The medium for 293T cells was replaced the next day, and cells were cultured for another 24-48h. Lentivirus-containing supernatants was collected and centrifuged at 3000 rpm for 15 minutes at 4° C to pellet debris. The virus supernatants were filtered through a 0.45μm filter and stored at -70 °C.

#### (ii) Titration of the lentivirus and transduction of target cells

Titration and transduction were done as described previously [37,38]. Briefly, 2×10^5^ 293T cells were plated in each well of a 6-well plate one day before infection. Three dilutions (1/10,000, 1/1,000, and 1/100) of the viral stocks containing full-length ORF of CXCL1 in the presence of transduction adjuvant polybrene (8μg/ml) were used to transduce the cells. The medium was changed 24h after transduction. Cells were removed 72h post-transduction and analyzed by flow cytometry for GFP expression. The titer was determined as follows: transduction units/ml = (average cell number at the time of transduction×% of GFP-positive cells) / 100× dilution factor. 2×10^5^ THP-1 cells were seeded in each well of a 6-well plate in 500μl of complete media and transduced by lentiviral vectors at a multiplicity of infection of 10:1 in the presence of polybrene (8μg/ml). Stable transfected single clone THP-1 cells with lentivirus containing CXCL1 (CXCL1-overexpressing THP-1 cells) was selected and was analyzed by flow cytometry and Real-time RT-PCR.

#### (iii) ELISA

Culture supernatants for transduced THP-1 cells were collected and was determined using a human CXCL1 ELISA kit (R&D systems). The assay was performed according to the manufacturer’s instructions.

### Plasmid construction and transfection

The full-length CXCR2 cDNA was obtained by RT-PCR from HBMEC, and was cloned into pcDNA3.1/myc-hisB using EcoRⅠand XbaⅠ. HBMEC were transient transfected with the constructed plasmids using Lipofectamine 2000 as the instruction and cells were conﬁrmed by Western blot analysis.

### TEER and HRP ﬂux measurement

TEER assay were performed as described previously [32]. 2 × 10^5^ HBMEC were seeded on the upper chamber of Transwell insert with 0.4μm pore size in 24-well plates. Experiments were conducted 4d after plating when TEER was > 180 Ω × cm^2^. CXCL1-overexpressing THP-1 cells (1 × 10^6^) were loaded into the upper chamber of the Transwell insert and Aβ (125nM) was added to the lower chamber. After incubation for 2, 4, 8, and 24h, TEER was measured using an EVOM voltohmmeter (World Precision Instruments, Sarasota, FL). The ﬁnal TEER values were calculated as ohm.cm^2^ by multiplying it with the surface area of the monolayer. For horseradish peroxidase (HRP) flux assay, after TEER was measured described above, Transwell inserts were transferred to a new 24-well plate. 0.5 μM HRP (Sigma-Aldrich, St. Louis, MO) in serum-free RPMI 1640 medium was added to the upper compartment of the Transwell system. After 1h, the media from the lower chamber were collected and the HRP content of the samples were assayed colorimetrically. The HRP ﬂux was expressed as nanomol passed per cm^2^ surface area. Inhibition experiments were performed as described previously [32]. Brieﬂy, HBMEC cultured in Transwell insert were pretreated with the inhibitors SP600125 (100 nM), Y27632 (10 μM), LY294002 (25 μM), Wortmannin (100 nM), Gö6976 (100 nM) and Gö6983 (100 nM), respectively, for 1h. And then culture medium was removed, CXCL1-overexpressing THP-1 cells were loaded into the upper chamber of Tranwell insert and Aβ (125nM) was added to the lower chamber. After incubation for 8h, TEER and HRP ﬂux measurement were carried as described above.

### Immunoﬂuorescence

CXCL1-overexpressing THP-1 cells were co-cultrued with HBMEC transduced with CXCR2 or vector control on sterilized coverslips for 24h. Glass coverslips were ﬁxed with 4% paraformaldehyde for 10min, and permeabilized with 0.1% Triton X-100 for 10min. After blocking with 5% BSA in PBS, the cells were incubated with ZO-1 antibody (Zymed Laboratories) overnight at 4°C and then incubated with Alexa 594-conjugated secondary antibody (Invitrogen, Carlsbad, CA) for 1h. The nuclei were stained with 4′-6-Diamidino-2-phenylindole (DAPI) (Sigma-Aldrich, St. Louis, MO). The cells were washed and the coverslips were mounted and analyzed using immunoﬂuorescence microscope (Olympus BX51, Tokyo, Japan).

### Rho activation assay

Rho activation assay kit (Upstate Biotechnology, Lake Placid, NY) was used to determine the activation of Rho proteins in HBMEC co-cultured with CXCL1-overexpressing THP-1 cells. Brieﬂy, cell lysates were incubated with rhotekin Rho-binding peptide immobilized on agarose beads for 45min, and activated GTP-Rho bound to rhotekin-agarose was detected by Western blot using rabbit anti-RhoA antibody (Santa Cruz Biotechnology).

### Statistical analysis

Data were presented as mean ± SD. Statistical signiﬁcance between two groups was analyzed by Student’s *t*-test. One-way ANOVA was used to compare multiple groups. A *P* value of <0.05 was considered signiﬁcant.

## Results

### 1. Up-Regulation of CXCL1 in AD patient’s monocytes compared to age-matched controls

**Figure 1:**
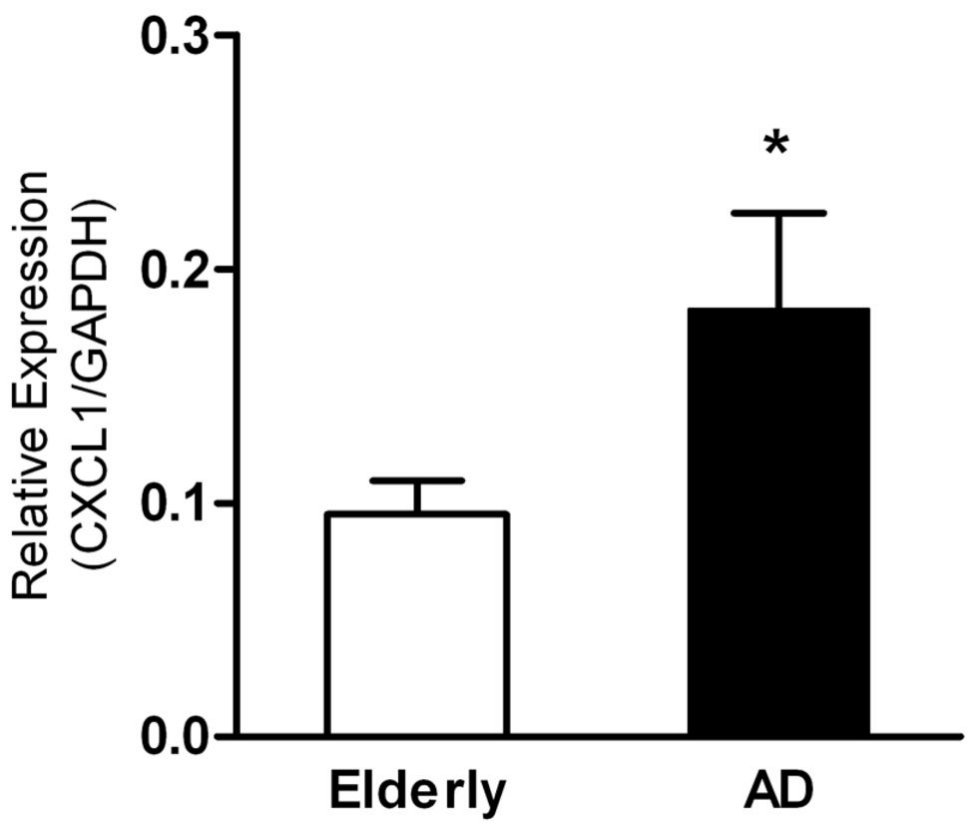
Monocytes derived from AD patients over-expressed CXCL1. Monocytes of 23 AD patients and 21 age-matched elderly controls were isolated and total RNA was extracted. Real-time RT-PCR was described in Material and Methods. CXCL1/GAPDH means the relative expression level of CXCL1 after correction for the expression of GAPDH. Data was the mean ± S.D. *p < 0.05 as compared with age-matched elderly controls.

In an attempt to identify the determinants that contributed to monocytes migrating from blood into brain in AD, we first performed microarray analysis of mRNA isolated from monocytes of 23 AD patients and 21 aged-matched elderly controls. Compared with aged-matched controls, the transcription of a few genes including CXCL1 in AD monocytes were up-regulated (>2.2 fold, *p*<0.001, data not shown). To confirm the transcriptional difference, Real-time RT-PCR was used to detect the mRNA expression of the CXCL1 in AD patient’s monocytes. The results showed that the expression of CXCL1 in AD patient’s monocytes was significantly higher than that in age-matched elderly controls (*p* < 0.01) (Figure 1).

### 2. Up-regulation of CXCL1 in monocytes from AD patients is associated with its enhanced ability to cross HBMEC monolayer in response to Aβ

**Figure 2:**
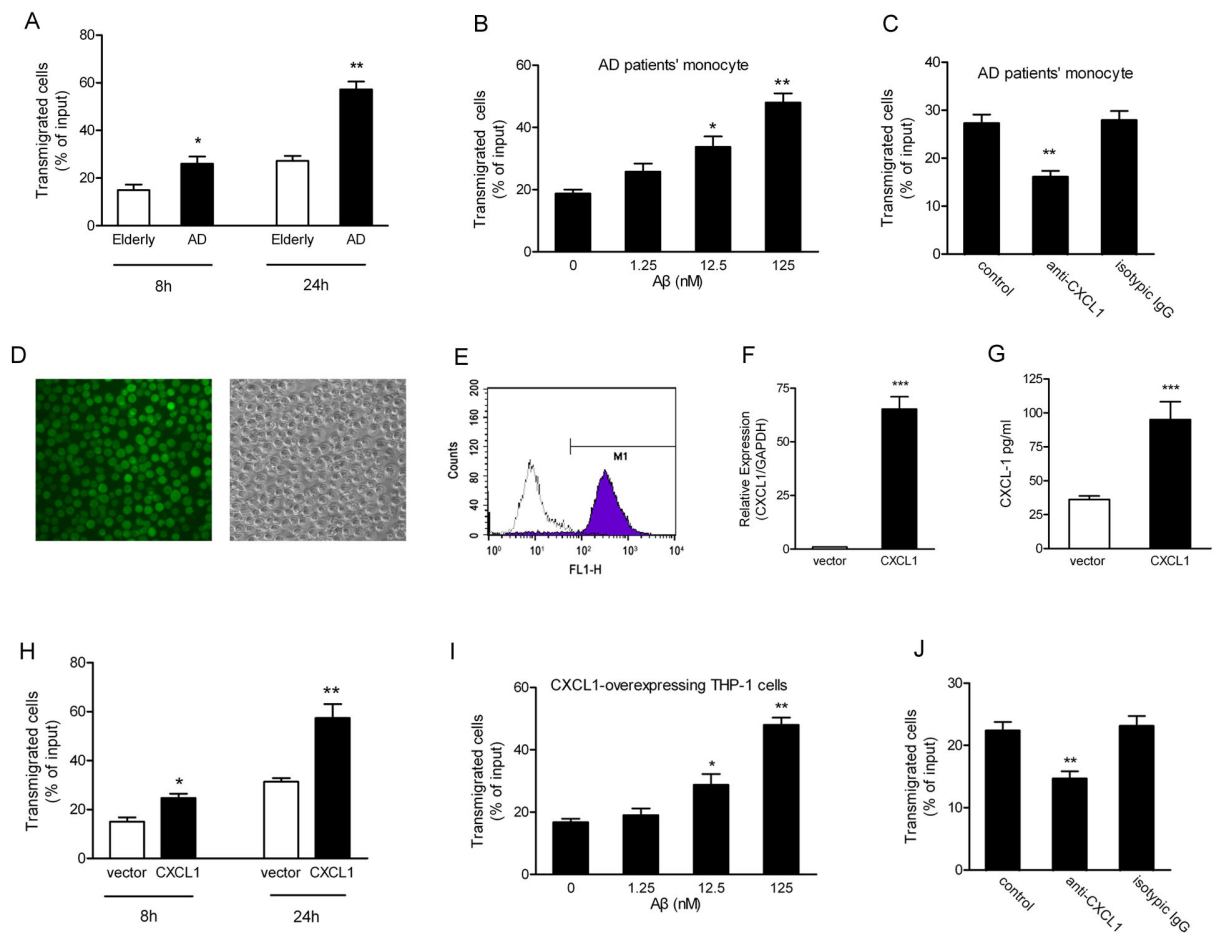
AD patient’s monocytes or CXCL1-overexpressing THP-1 cells had an enhanced ability to cross HBMEC monolayer in response to Aβ. (A) 125nM Aβ were added to the lower chamber of the Transwell insert cultured with HBMEC, AD patients’ monocytes were loaded on upper chamber of the Transwell insert and incubated for 8h or 24h. The cells transmigrated into the lower chamber were harvested and counted. Data were the mean±S.D. *p < 0.05, **p < 0.01 as compared with age-matched elderly controls (n=8); (B) Transendothelial migration assays were performed in the presence of Aβ in 0, 1.25, 12.5, and 125nM respectively in the lower chamber of the Transwell insert cultured with HBMEC. Monocytes derived from AD patients were added to the upper chamber of Transwell insert for 24h. Transmigrated cells were harvested and counted. The results represent three independent experiments. Data were the mean±S.D. *p < 0.05, **p < 0.01; (C) AD patients’ monocytes with anti-CXCL1 neutralizing antibody were loaded into the upper chamber. The cells transmigrated into the lower chamber were counted after 24h. Data were mean±S.D. **p < 0.01. (D) THP-1 cells was transduced by lentivirus based vectors and efficiently expressed GFP. (E) 1×10^6^ transduced THP-1 cells were analyzed by flow cytometry and GFP fluorescence intensity was measured on the FL1 channel. Approximately 97% of the THP-1 cells were GFP-positive. Real-time RT-PCR (F) or ELISA (G) was done to detect the expression of CXCL1 in THP-1 cells transduced with lentivirus based CXCL1. (H) Transendothelial migration assays were performed in the presence of Aβ in 125nM in the lower chamber of the Transwell insert cultured with HBMEC. CXCL1-overexpressing THP-1 cells were added to the upper chamber of Transwell insert for 8h or 24h. (I) In the presence of Aβ in 0, 1.25, 12.5, and 125nM respectively in the lower chamber of the Transwell insert cultured with HBMEC, CXCL1-overexpressing THP-1 cells was added to the upper chamber of Transwell insert for 24h. (J) CXCL1-overexpressing THP-1 cells with anti-CXCL1 neutralizing antibody were loaded into the upper chamber in the presence of Aβ in 125nM in the lower chamber of the Transwell insert cultured with HBMEC. The cells transmigrated into the lower chamber were counted after 24h. Transmigrated cells were harvested and counted. The results represented three independent experiments. Data was the mean±S.D. *p < 0.05, **p < 0.01.

HBMEC monolayer cultured on Transwell insert has been broadly used as a BBB model [39]. To mimic AD patient brain’s microenvironment, Aβ was added to lower chamber of Transwell insert and an Aβ-containing in vitro BBB model was constructed. Monocytes derived from AD patients were added to the upper chamber of Transwell insert. The results showed that when Aβ (125 nM) was added to the lower chamber for 8h, the migration rate of monocytes from AD patient had an obvious elevation compared to the elderly aged-matched controls (Figure 2A). Furthermore, AD patients’ monocytes transendothelial migration had an obvious increase for 24h with extension of the incubation period although a slight elevation was detected in the aged-matched controls. Meanwhile, AD patients’ monocytes transendothelial migration was increase in a dose-dependent (Figure 2B) under an Aβ-containing in vitro BBB model condition. Monocyte constitutively expresses several chemokine or chemotactic cytokines, which are involved in leukocyte migration [40]. In order to identify whether CXCL1 was a determinant contributed to AD patients’ monocytes migration, neutralizing experiments were carried. The result showed that the anti-CXCL1 antibody effectively blocked AD patients’ monocytes migration through the in vitro BBB model, when Aβ was added to the lower chamber (Figure 2C).

Next, we established a CXCL1-overexpressing monocytic THP-1 cells line using a lentiviral-based vector. Lentivirus containing full-length ORF of CXCL1 and a GFP tag was able to transducer into THP-1 cells (Figure 2D). Positive THP-1 cells were selected and analyzed for GFP expression using flow cytometry (Figure 2E). The lentiviral vectors were able to consistently transduce 97.5% of target cells. Transduced cells have a significantly high level expression of CXCL1 examined by Real-time RT-PCR (Figure 2F) and ELISA (Figure 2G). Next, we detected the migration of CXCL1-overexpressing THP-1 cells across the Aβ-containing in vitro BBB model. The results showed that the migration rate of CXCL1-overexpressing THP-1 cells had an obvious elevated migration across HBMEC monolayer in a time- (Figure 2H) and dose-(Figure 2I) dependent manner in response to Aβ stimulation. Neutralizing experiments result showed that the anti-CXCL1 antibody effectively blocked CXCL1-overexpressing THP-1 cells migration through the in vitro BBB model, when Aβ was added to the lower chamber (Figure 2J). These data indicated that CXCL1 played an important role in Aβ dependent monocytes transendothelial migration across HBMEC monolayer in vitro.

### 3. Bone marrow-derived microglia accumulation in APP mouse brain could be blocked by anti-CXCL1 antibody

**Figure 3:**
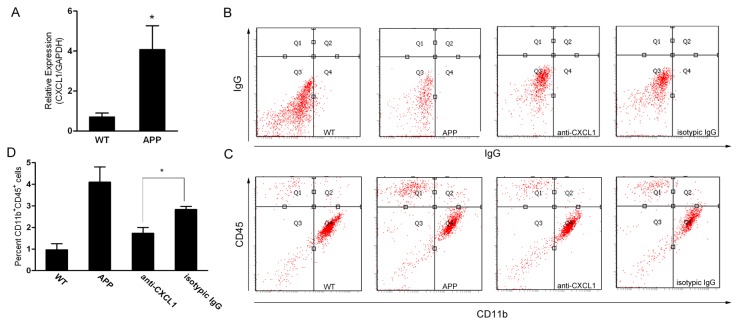
Flow cytometry analysis of microglial accumulation in APP mouse. (A) Monocytes of APP/PS1 tansgenetic mouse and wild type controls were isolated and total RNA was extracted. Real-time RT-PCR was described in Material and Methods. CXCL1/GAPDH means the relative expression level of CXCL1 after correction for the expression of GAPDH. Data was the mean ± S.D. *p < 0.05. (B) and (C) representative dot plots for ﬂow cytometry of CD11b and CD45 stained brain cells. (D) Quantitation of the percent of total brain cells positive for both CD11b and CD45 (CD11b^+^CD45^+^) by ﬂow cytometry (n = 3 mice per group, *p < 0.05).

In order to further identify whether CXCL1 was a determinant for monocytes across migrating from blood into brain in vivo, APP/ presenilin 1 (PS1) transgenic mouse were used as a mouse model of AD. Monocytes from peripheral blood were isolated, and Real-time RT-PCR was used to detect the mRNA expression of the CXCL1 in APP mouse monocytes. The result showed that the expression of CXCL1 in APP mouse monocytes was significantly higher than wide type mouse (*p* < 0.05) (Fig. 3A). Next, ﬂow cytometry, with antibodies to CD11b and CD45 as described [26,41,42] was used to determine the number of microglia accumulating in the brains Using this method, mononuclear cells recruited from the peripheral blood were identiﬁed as a CD11b^+^CD45^hi^ population of cells, whereas resident microglia are distinguished as a CD11b^+^CD45^lo^ population of cells [26,41,42]. Whole brains from APP mice showed a marked 4.24-fold increase in the percentage CD11b^+^CD45^hi^ cells compared to wild-type brains (4.1% versus 0.96%, respectively) (Figure 3B–D). This increase in CD11b^+^CD45^hi^ cells was markedly attenuated when the APP mouse were injected with anti-CXCL1 antibody, whereas there were no significant differences between the APP group and the isotypic IgG group, although slight decreases in isotypic IgG group were detected (Figure 3B–D). Our data indicate that CXCL1 plays an important role in the accumulation of bone marrow-derived microglia in the brain of APP mice.

### 4. Up-regulation of CXCR2 expression on HBMEC by Aβ stimulation contributed to CXCL1-overexpressing THP-1 cells transendothelial migration

**Figure 4:**
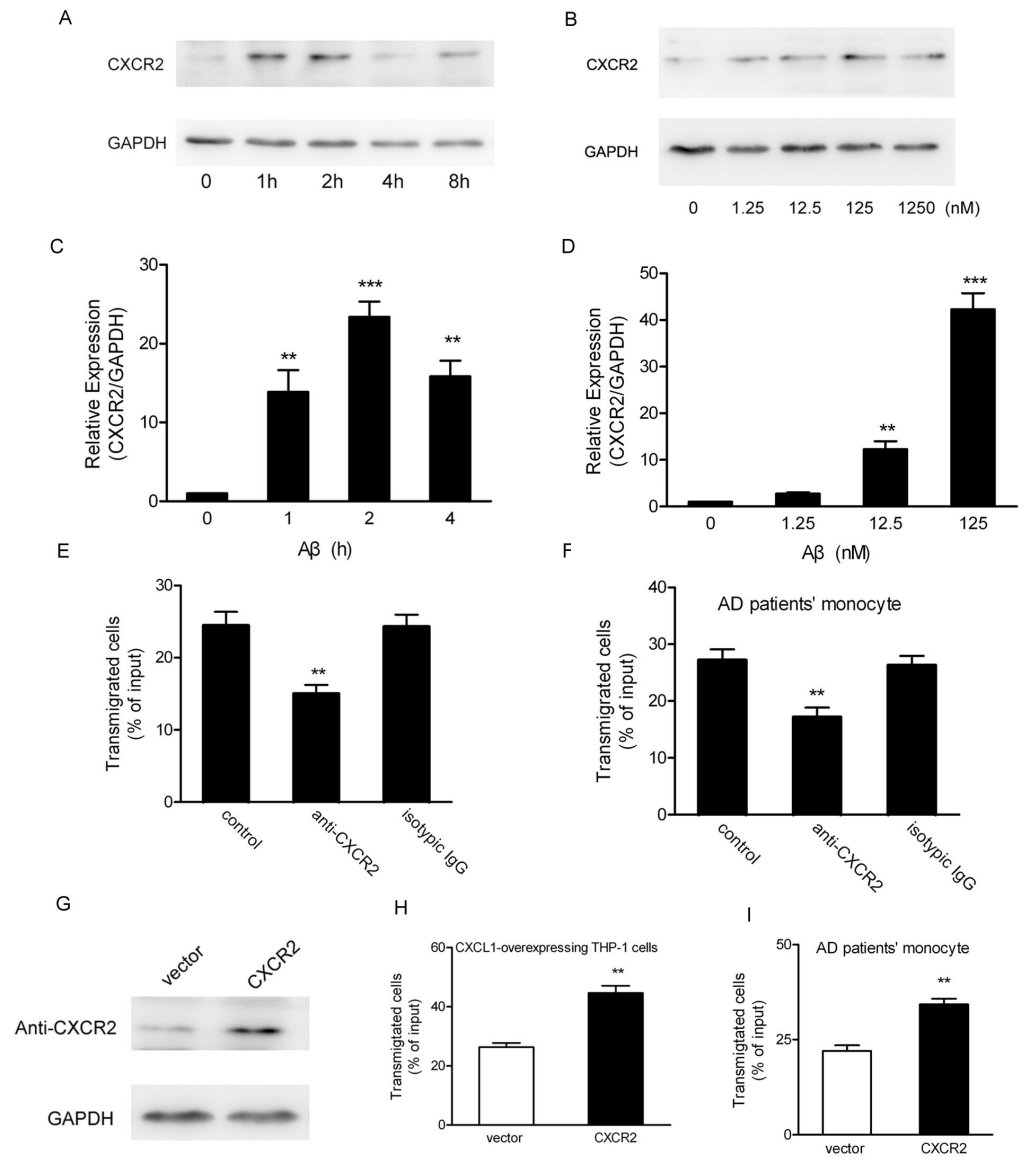
CXCR2 in HBMEC was up-regulated by Aβ sitmulation and contributed to CXCL1-overexpressing THP-1 cells transendothelial migration. (A) HBMEC were exposed to Aβ for indicated time at a concentration of 125 nM or (B) indicated concentrations for 1h. CXCR2 expression on HBMEC was detected by Western blot using anti- CXCR2 antibody. Untreated HBMEC were used as control. (C) HBMEC were incubated with Aβ for indicated times at a concentration of 125 nM, or with indicated concentrations for 1h (D), and the total RNA of HBMEC was extracted for real-time RT-PCR. Expression was normalized to GAPDH and the group without Aβ was set to 1. Data were the mean ± SD (n=3). *p < 0.05. The HBMEC monolayer cultured on the Transwell insert was placed on a 24-well plate while Aβ was present in the lower chamber. CXCL1-overexpressing THP-1 cells（E) or AD patients’ monocytes（F) with anti-CXCR2 neutralizing antibody were loaded into the upper chamber. The cells transmigrated into the lower chamber were counted after 24h. Data were mean±S.D. **p < 0.01. (G) The HBMEC expressing full-length ORF of CXCR2 by transient transfection was identiﬁed by Western blot using anti-CXCR2 antibody. HBMEC transfected with vector were used as control. CXCL1-overexpressing THP-1 cells (H) or AD patients’ monocyts (I) were loaded into upper chamber of Transwell insert cultured with HBMEC transfected with CXCR2. The transmigrated monocytes were counted. Data were the mean ± S.D. *p < 0.05, **p < 0.01.

Chemokines are chemotactic cytokines that bind specific G protein-coupled receptors on the surface of cells and mediate the accumulation of leukocytes under physiologic and pathologic conditions [40]. CXCR2 is the only identified receptor for CXCL1 [43]. In AD brain, CXCR2 appears to be the most strongly expressed and was also strongly up-regulated in a subpopulation of neuritic plaques [44]. However, due to the complex tight junctions between brain endothelial cells and the lack of pinocytotic activity of the brain endothelial cells prevent the diffusion of chemokines across the BBB [45]. Given the fact that the migration rate of CXCL1-overexpressing THP-1 cells was elevated using in vitro BBB model containing Aβ, and CXCR2 was expressed on HBMEC at low levels (Figure 4A). We tried to test whether there was an Aβ-related up-regulation of CXCR2 expression in HBMEC. The result showed that Aβ exerted a time- (Figure 4A and C) and dose- dependent (Figure 4B and D) effect on CXCR2 mRNA and proteins levels expression when HBMEC were exposed to Aβ. Furthermore, results of neutralizing experiments showed that the anti-CXCR2 antibody effectively blocked CXCL1-overexpressing THP-1 cells (Figure 4E) and AD patients’ monocytes (Figure 4F) migration through the in vitro BBB model, when Aβ was added to the lower chamber. Next, we explored the effect of CXCR2 over-expressing on HBMEC in monocytes transendothelial migration. The results showed that a high level expression of CXCR2 (Figure 4G) in HBMEC signiﬁcantly promoted the CXCL1-overexpressing THP-1 cells transendothelial migration (Figure 4H). In addition, AD patients’ monocytes had a significant higher migration rate when the upper chamber of Transwell insert were seeded with a high level expression CXCR2 in HBMEC (Figure 4I). As proved by our data above, up-regulation of CXCR2 expression on HBMEC by Aβ stimulation was likely to interact with its ligand CXCL1 to promote the monocytes transendothelial migration.

### 5. CXCL1-overexpressing THP-1 cells induces the disassembly of tight junction between brain endothelium over-expressed CXCR2

**Figure 5:**
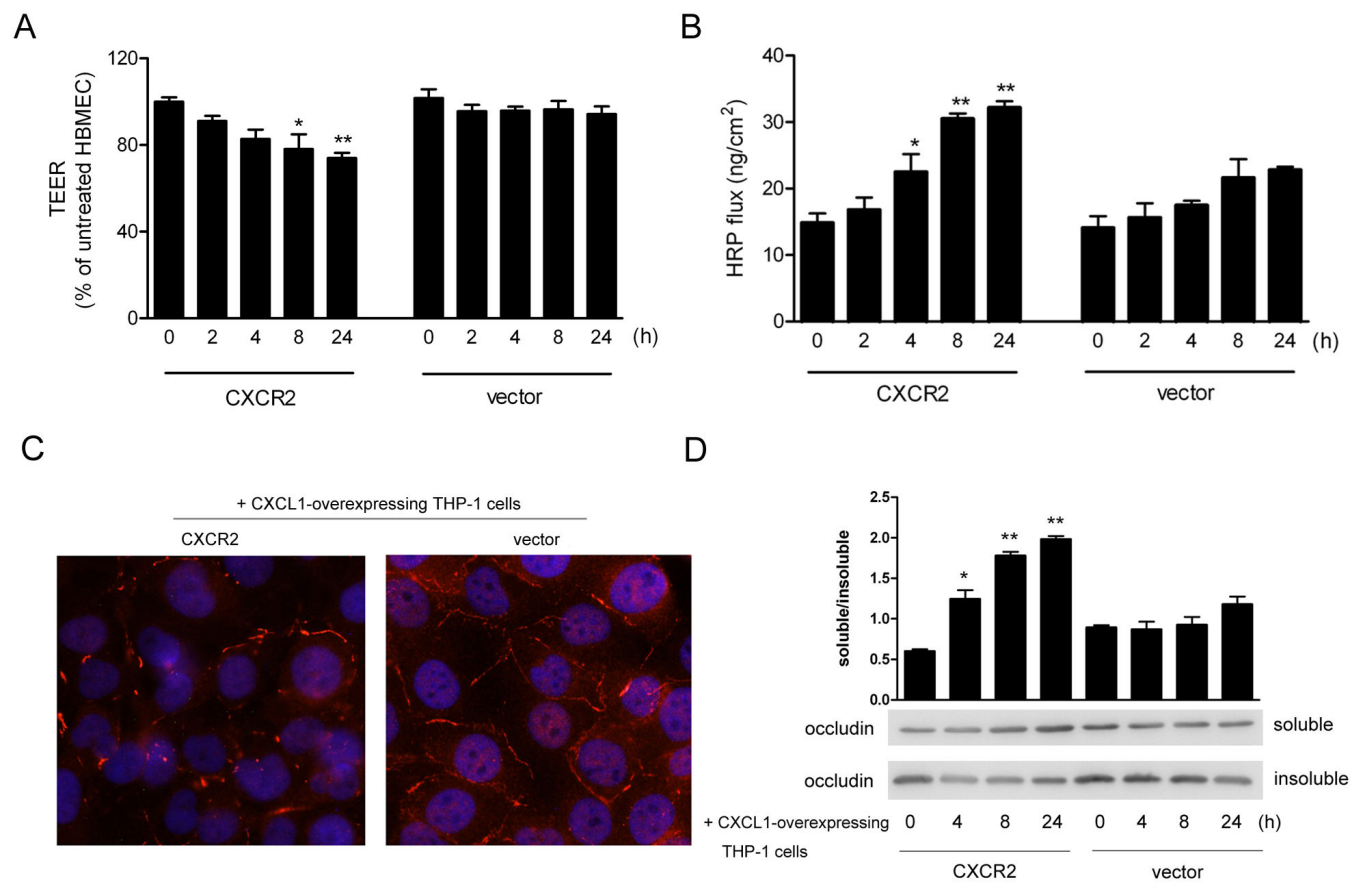
Disassembly of tight junction between brain endothelium over-expressed CXCR2 were induced by CXCL1-overexpressing THP-1 cells. (A) HBMEC transfected with CXCR2 or vector control was seeded into the upper chamber of Transwell insert until they reached conﬂuence. CXCL1-overexpressing THP-1 cells was added to the upper chamber of Transwell insert for the indicated time. Then the TEER was measured by EVOM volt-ohmmeter. The TEER value was expressed as the percent of the 0h. *p < 0.05, **p < 0.01. (B) HBMEC transfected with CXCR2 or vector control was seeded into the upper chamber of Transwell insert until they reached conﬂuence. CXCL1-overexpressing THP-1 cells was added to the upper chamber of Transwell insert for the indicated time. The medium in the upper chamber was replaced by RPMI-1640 containing 0.5 μM of HRP, and then the medium in the lower chamber was collected and the HRP concentration was assessed. Data were means ± S.D. of three independent experiments. *p < 0.05, **p < 0.01. (C) CXCL1-overexpressing THP-1 cells was co-cultrued with HBMEC transfected with CXCR2 or vector control on sterilized coverslips for 24h. The distribution of ZO-1 in the HBMEC was visualized by immunoﬂuorescence. Nuclei were counterstained with DAPI. Scale bar: 40 μm. (D) CXCL1-overexpressing THP-1 cells was co-cultrued with HBMEC transfected with CXCR2 or vector control for the indicated time. The soluble and insoluble forms of occludin were analyzed by Western Blot. *p < 0.05, **p < 0.01.

BBB restricts the movement of soluble protein and immune cells from periphery into CNS, which is mostly due to the tight junction between adjacent endothelial cells, conferring low paracellular permeability and high electrical resistance [46]. In order to investigate whether there was a dysfunction for tight junction between brain endothelium induced by CXCL1-overexpressing THP-1 cells, the permeability of HBMEC monolayer were examined by measuring TEER and paracellular HRP ﬂux after CXCL1-overexpressing THP-1 cells interaction with HBMEC transfected with CXCR2. The results showed that, compared to the controls, CXCL1-overexpressing THP-1 cells induced a decrease of TEER in the HBMEC transfected with CXCR2 (Figure 5A), meanwhile, an increase of HRP ﬂux in a time-dependent manner was observed (Figure 5B). Tight junction proteins were composed of transmembrane integral proteins including claudins, occludin, and several cytoplasmic accessory proteins such as ZO-1 and ZO-2.They played an important role in maintaining the integrity of tight junction. Next, we detected the changes of tight junction proteins in the process. The detergent-insoluble occludin was an indicator of tight junction integrity and once tight junction was weakened, occludin relocated to the detergent-soluble fraction in cells [47]. As shown in Figure 5D, there was an obvious shift in occludin distribution from insoluble to soluble fractions prepared from HBMEC co-cultured with CXCL1-overexpressing THP-1 cells as the indicated time. Meanwhile, the distribution of tight junction proteins was visualized by immunoﬂuorescence. The results showed when the HBMEC transfected with vector were co-cultured with CXCL1-overexpressing THP-1 cells, a characteristic polygonal shape and linear pattern of immuno-staining for ZO-1 at cell-cell borders were observed, whereas in the HBMEC transfected with CXCR2 co-cultured with CXCL1-overexpressing THP-1 cells, continuous lines of ZO-1 staining gradually became discontinuous, segmented and dotted (Figure 5C). These data suggested that tight junction disassembly in HBMEC were correlated with CXCL1-overexpressing THP-1 cells interaction with CXCR2 in HBMEC.

### 6. CXCL1-overexpressing THP-1 cells transendothelial migration induced by Aβ was associated with Rho/ROCK signalling activation

**Figure 6:**
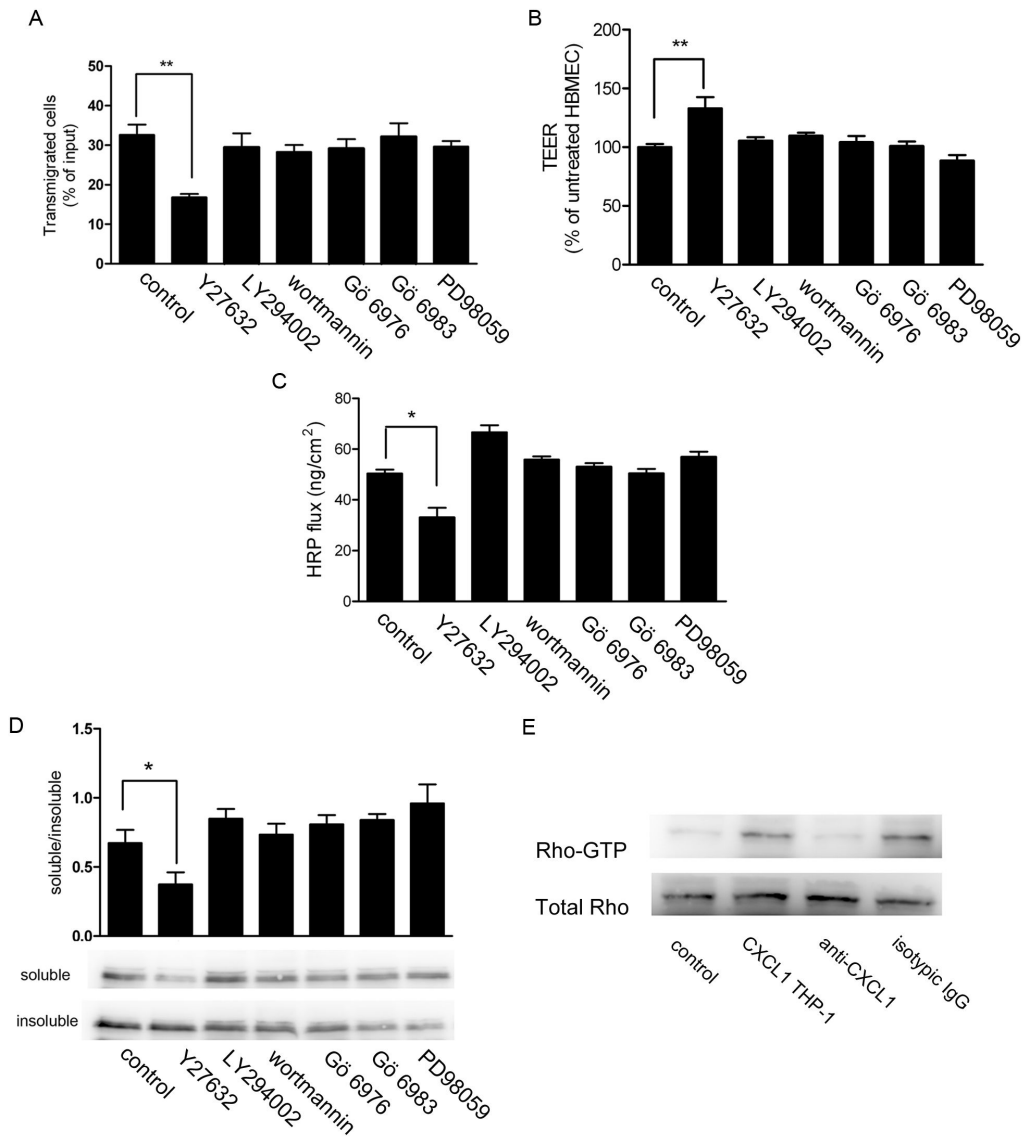
ROCK inhibitor Y27632 could block CXCL1-overexpressing THP-1 cells transendothelial migration. HBMEC were pretreated with speciﬁc inhibitors as described in Material and Methods and then co-cultured with CXCL1-overexpressing THP-1 cells for 24h. Transmigrated CXCL1-overexpressing THP-1 cells (A), TEER (B) and HRP ﬂux (C) were measured, respectively. The different detergent solubility of endothelial occludin were analyzed by Western blot (D). Data were means ± S.D. of three independent experiments. *p < 0.05, **p < 0.01. (E) Conﬂuent HBMEC was co-cultured with CXCL1-overexpressing THP-1 cells for 24h. The Rho activity in HBMEC was analyzed.

Several different types of intracellular signalling pathways have been implicated to participate in the regulation of endothelial permeability. To investigate which signaling pathway in HBMEC might be associated with CXCL1-overexpressing THP-1 cells transendothelial migration induced by Aβ, the effects of speciﬁc inhibitors for the signaling pathways were tested. As shown in Figure 6A, only ROCK inhibitor, Y27632, could block CXCL1-overexpressing THP-1 cells transendothelial migration, whereas other inhibitors had no effects. Similar results were obtained from the TEER assay (Figure 6B), HRP ﬂux (Figure 6C) and the detergent solubility of occludin (Figure 6D). These ﬁndings suggested that ROCK other than signaling pathways was involved in CXCL1-overexpressing THP-1 cells transendothelial migration. To further conﬁrm the inhibition experiments, we detected the activity status of signaling molecules in HBMEC co-cultured with CXCL1-overexpressing THP-1 cells. Because the ROCK activation is mostly regulated by the small GTPase RhoA, the activity of endothelial RhoA protein was investigated by affinity precipitation. Figure 6E revealed that CXCL1-overexpressing THP-1 cells induced activation of RhoA at 8h, whereas the alteration of RhoA activity was abolished when CXCL1-overexpressing THP-1 cells were treated with anti-CXCL1 antibody. These results strongly supported that Rho/ROCK signalling activation was involved in Aβ induced CXCL1-overexpressing THP-1 cells transendothelial migration.

## Discussion

Bone-marrow-derived cells have the ability to populate the CNS and differentiate into functional parenchymal microglia as well as perivascular microglia especially in several neuro-inflammatory conditions, such as bacterial meningitis, primary demyelination of CNS and Parkinson’s disease [48,49]. Recently, many evidence has demonstrated that bone-marrow-derived microglia, which most derived from peripheral monocytes, have a preferentially attachment to amyloid deposition in rodent transgenic models with AD [4,22–26]. However, the mechanism contributed to monocytes crossing the BBB remains largely unknown. Our present study showed for the first time that, CXCL1 is over-expressed in AD patients’ monocytes, which contributes to Aβ-induced transendothelial migration of monocytes through the interaction with CXCR2 in HBMEC.

CXCL1, also called growth-related oncogene-α (GRO-α), belongs to the CXC group of chemokines. This chemokine was initially cloned from fibroblasts, and expressed by macrophages, neutrophils and epithelial cell [50]. CXCL1 regulates growth of melanocytes, acts as a chemoattractant for leukocytes recruitment and correct positioning of cells in tissues [51]. Under some pathologic conditions, such as Atherosclerosis [52], CXCL1 trigger monocytes adhesion and recruitment to endothelium lesion. In AD, Xia et al [53] reported that CXCL1 could work as a potent trigger induces hypermethylation of tau in mouse primary cortical neurons through ERK1/2 and PI3K pathway. Recent study showed that CXCL1 could contribute to inflammation response in the development of AD, but not as a potential genetic factor conferring the predisposition to AD in the pathogenesis of this disease [54]. We show here that there is signiﬁcantly higher CXCL1 expression in peripheral monocytes of AD patients than age-matched control subjects. To explore the signiﬁcance of CXCL1 over-expression in peripheral monocytes derived from AD patients, we prepared the in vitro BBB model mimicking the microenvironment of AD brain. We found that AD patients’ monocytes had a signiﬁcantly higher capability to transmigrate the HBMEC monolayer, which displayed Aβ related time- and dose- dependence. In order to further identify the role of CXCL1, monocytic THP-1 cell line with stable transfection of CXCL1 was established. We found that CXCL1-overexpressing THP-1 cells have a similar higher capability to transendothelial migration under an Aβ-containing in vitro BBB model. Further neutralizing antibody for CXCL1 effectively blocked AD patients’ monocytes or CXCL1-overexpressing THP-1 cells transendothelial migration. Importantly, monocytes derived from APP/PS1 transgenic mouse also expressed signiﬁcantly higher CXCL1. CD11b^+^CD45^hi^ population of cells that were recruited from the peripheral blood were markedly impairs accumulation in APP mouse brain by anti-CXCL1 antibody. These results indicated that high level of CXCL1 expression in monocytes may be one reason why increased occurrence of monocytes was found in the brains of transgenic mice with AD, and we identified another role of CXCL1 in AD pathogenesis.

Aβ plays a central role in the development of AD with an increased deposition in both brain parenchyma and cerebral vasculature [55]. Aβ protein may active endothelial monolayers and induced migration of monocytes across HBMEC in vitro though relatively the mechanism by which Aβ could induce these events is not clear [56]. We also obtained the similar results that Aβ triggered the monocytes crossing HBMEC. However, why do CXCL1-overexpressing THP-1 cells have a higher migrated ability across an Aβ-containing HBMEC monolayer? It is known that CXCL1 is small chemokines. Chemokines is chemotactic cytokines that bind its receptor on the surface of cells and mediate the accumulation of the leukocytes at sites of inflammation. For example, CCL2 is a potent monocyte chemoattractant through binding its receptor CCR2 to induce monocyte arrest on endothelium [57,58]. In AD, CCL2-CCR2 interactions seem to play a key part in recruitment and/or activation of microglia to sites of Aβ deposition in AD [59]. In our previous study [31,60], we also found that chemokines interacted with its receptors plays an important role in immune cells transendothelial migration and recruitment into CNS in AD model rats. The only identified receptor for CXCL1 is CXCR2 (IL-8RB). In vitro cultured mouse brain microvascular endothelium cells constitute express CXCR2 [61]. In our study, we detected low expression level of CXCR2 in cultured HBMEC. It should be noted that there was a significant increased expression of CXCR2 when HBMEC were stimulated with Aβ, even displayed a time- and dose- dependence manner. In the AD brain, Xia et al. investigated CXCR2 were the most strongly expressed and was also strongly up-regulated in a subpopulation of neuritic plaques. Therefore, it is likely that CXCR2 is an AD-related gene change its expression with Aβstimulation and deposition. The interaction between CXCL1 and CXCR2 is critical for mediating leukocyte recruitment in many diseases such as Lyme Arthritis, Carditis and experimental brain abscesses. Next, we try to explore the potential novel role of CXCR2 on brain endothelial cells, that is, whether CXCL1 interacted with CXCR2 contributes to monocyte transendothelial migration. We found that CXCL1-overexpressing THP-1 cells have more than 50% migrated rate across the transfected with CXCR2 HBMEC compared to the controls. These data is the first to suggest that AD monocytes transendothelial migration occurs as the result of AD monocyte up-regulated CXCL1 interactions with receptor CXCR2 on HBMEC.

Tight junction between adjacent endothelial cells with low paracellular permeability and high electrical resistance is the main characteristics for BBB [46]. Our results showed that when CXCL1-overexpressing THP-1 cells were co-cultured with HBMEC transfected with CXCR2, it may cause an apparent disruption in the distribution of ZO-1. It is consistent with our observation that CXCL1-overexpressing THP-1 cells increased the paracellular permeability of HBMEC monolayer transfected with CXCR2. These results suggested that the interaction of CXCL1-overexpressing THP-1 cells and CXCR2 expressed in HBMEC may trigger the tight junction ‘opening’ and contributed to monocytes transendothelial migration. Our results also showed that CXCL1-overexpressing THP-1 cells interacted with HBMEC triggered endothelial tight junction “opening” via a signaling molecule ROCK, which play an important role in tight junction “opening”. Our previous study reported that MIP-1α derived from AD patients T cell interacted with CCR5 expressed on HBMEC was involved in T cell transendothelial migration. It is suggested that chemokines and its receptor plays a key role in AD patient immune cells recruitment and accumulation through regulated the brain endothelial tight junction.

In summary, our findings indicated that monocytes derived from AD patients overexpressing CXCL1, which is a determinant for Aβ-induced transendothelial migration. CXCL1 expressed by monocytes and CXCR2 on HBMEC is involved in monocytes migrating from blood to brain in AD patients. Further studies are needed to determine the molecular mechanism for the up-regulation of CXCL1 in AD patients’ monocytes.
